# Protective Effect of Garlic on Cellular Senescence in UVB-Exposed HaCaT Human Keratinocytes

**DOI:** 10.3390/nu8080464

**Published:** 2016-07-29

**Authors:** Hye Kyung Kim

**Affiliations:** Department of Food & Biotechnology, Hanseo University, Haemi-Myun, Seosan, Chungnam 356-706, Korea; hkkim111@dreamwiz.com; Tel.: +82-41-660-1454

**Keywords:** garlic, UVB irradiation, HaCaT cells, senescence, MMP-1, SA-β-gal, SIRT1, pro-inflammatory cytokine

## Abstract

Ultraviolet (UV) irradiation generates reactive oxygen species (ROS) in the cells, which induces the cellular senescence and photoaging. The present study investigated the protective effects of garlic on photo-damage and cellular senescence in UVB-exposed human keratinocytes, HaCaT cells. An in vitro cell free system was used to examine the scavenging activity of 2,2-diphenyl-1-picrylhydrazyl (DPPH) free radicals and nitric oxide (NO). The effect of garlic extract on ROS formation, MMP-1 protein and mRNA expressions, cytokines such as interleukin (IL)-1β and IL-6, senescence associated-β-galactosidase (SA-β-gal) activity, and silent information regulator T1 (SIRT1) activity were determined in UVB-irradiated HaCaT cells. Garlic exhibited strong DPPH radical and NO scavenging activity in cell free system exhibiting IC_50_ values of 2.50 mg/mL and 4.38 mg/mL, respectively. Garlic pretreatment attenuated the production of UVB-induced intracellular ROS. MMP-1 level, which has been known to be induced by ROS, was dramatically elevated by UVB irradiation, and UVB-induced MMP-1 mRNA and protein expressions were significantly reduced by garlic treatment (50 µg/mL) comparable to those of UV-unexposed control cells. UV-induced pro-inflammatory cytokine productions (IL-6 and IL-1β) were significantly inhibited by pretreatment with garlic in a dose-dependent manner. SA-β-gal activity, a classical biomarker of cellular senescence, and SIRT1 activity, which has attracted attention as an anti-aging factor in recent years, were ameliorated by garlic treatment in UV-irradiated HaCaT cells. The present study provides the first evidence of garlic inhibiting UVB-induced photoaging as a result of augmentation of cellular senescence in HaCaT human keratinocytes.

## 1. Introduction

Aging is due to an accumulation of detrimental changes overtime at both the molecular and cellular levels, which ultimately leads to a functional decline at the tissue and organ level. Although the etiology of aging has not been fully understood, several cellular and molecular hallmarks may contribute to this process. Cellular senescence can be induced by telomere shortening, activation of tumor suppressor genes and oncogenes, chronic inflammation, oxidative stress, and ultraviolet (UV) irradiation [1,2]. Cells undergoing senescence are associated with a rise in intracellular reactive oxygen species (ROS) [3], as well as hyperactivity of the transcription factor nuclear factor (NF)-κB and over-expression of inflammatory cytokines such as TNF-α, IL-1β, and IL-6 [4,5]. Therefore, oxidative stress and chronic inflammatory process are major risk factors underlying aging and age-related diseases [3,6].

Aging of the skin is a complex biological phenomenon consisting of two components: intrinsic aging and photoaging caused by environmental exposure, primarily to UV radiation [7]. UV irradiation is the most common environmental factor that damages the human skin, leading to conditions such as skin carcinogenesis, inflammation, solar erythema and premature senescence [7]. Excessive exposure to UV irradiation generates ROS in skin [8], and leads to the activation of transcription factors that induces the expression of pro-inflammatory cytokines and metalloproteinase (MMPs) [7] resulting cellular senescence. Collagen-degrading MMP-1 is upregulated and serves as the primary MMP in UV-exposed skin [7]. Therefore, excessive degradation of collagen and matrix by UV-induced MMPs is a characteristic feature of photo-damaged skin, and MMP is used as a major marker of UVB-induced photoaging as well as skin inflammation.

For a number of years, the occurrence of senescence in vivo has been questioned, due mainly to the lack of specific markers. In 1995, Dimri and colleagues reported that several types of human senescent cells expressed a β-galactosidase that was detectable by a histochemical assay [9]. Senescence-associated β-galactosidase (SA-β-gal) is a hydrolase enzyme that catalyzes the hydrolysis of β-galactosides into monosaccharides only in senescent cells, and age-dependent increase in SA-β-gal staining was observed in human skin [9].

Sirtuins (silent mating type information regulation 2 homolog, SIRTs) are a family of NAD(+)-dependent enzymes and well-known modulators of lifespan in many species [10]. SIRTs have a role in gene repression, metabolic control, apoptosis and cell survival, DNA repair, development, inflammation, and healthy aging [11,12]. To date, seven human sirtuins have been identified and named SIRT1-7 [13], and expression of all seven sirtuins has been demonstrated in human epidermal and dermal cells [14]. SIRT1 is the most extensively studied human sirtuin, and UV irradiation downregulates SIRT1 expression in cultured skin keratinocytes [15].

Garlic has long been used widely not only as a flavoring agent but also as a folk medication and is one of the most well-known herbal medicines worldwide. Numerous therapeutic effects of garlic and its constituents such as antioxidant, anti-microbial, anti-atherosclerotic, anti-diabetic, anti-mutagenic, anti-carcinogenic and immunomodulatory activities have been reported [16,17,18,19,20]. However, the effects of garlic on anti-senescence activity in photoaging process have not yet been elucidated. The present study examined the inhibitory activity of garlic against cellular senescence and skin aging in the UV-exposed human keratinocyte cell line, HaCaT.

## 2. Materials and Methods

### 2.1. Preparation of Garlic Extract

Fresh garlic (*Allium sativum* L.), purchased from Seosan (Chungnam, Korea) in July 2012, were peeled, vacuum dried, and powdered. The samples were extracted with 80% ethanol at 65 °C for 5 h, filtered through a 0.45 µm filter (Osmonics, Minnetonka, MN, USA), and lyophilized.

### 2.2. Antioxidant Activity

Antioxidant activities in a cell free system were evaluated by free radical scavenging capacity and nitic oxide (NO) scavenging activity. The free radical scavenging activity of garlic extracts on 2,2-diphenyl-1-picrylhydrazyl (DPPH) radicals was determined using the method described by Huang et al. [21] with slight modification. Briefly, DPPH ethanol solution was added to various concentrations of garlic extract (0.4–50 mg/mL) in 96-well plates. After 30 min incubation at room temperature in the dark, the absorbance at 515 nm was measured by a plate reader (BioTek Inc., Winooski, VT, USA). The free radical scavenging activity of the sample was calculated by the following formula:

DPPH free radical scavenging activity (%) = (1 − As/Ab) × 100

where As is the absorbance of the sample and Ab is the absorbance of the blank.

NO production was assessed by measuring the nitrite content. Briefly, Griess reagent (0.1% N-1-naphthylenediamine dihydrochloride and 5% H_3_PO_4_ solution) was added to garlic extracts in a 1:1 (*v*/*v*) manner. After gentle mixing and 15 min incubation in the dark, NO levels were subsequently measured and compared with a standard curve. Absorbance values at 560 nm were measured using a microplate reader. The NO scavenging activity of the sample was calculated using the same formula as used for the DPPH scavenging activity. The IC_50_ values of DPPH and NO radical scavenging activities were obtained using GraphPad Prism 6 (La Jolla, CA, USA).

### 2.3. Cell Culture and UV Irradiation

An immortalized human keratinocyte cell line, HaCaT, obtained from the American Type Culture Collection (Rockville, MD, USA), was cultured in Dulbecco’s Modification of Eagle’s Medium (DMEM) containing 10% heat-inactivated FBS and 1% antibiotic-antimycotic (GIBCO-BRL, Grand Island, NY, USA) at 37 °C under a humidified atmosphere of 95% air and 5% CO_2_. Cells were treated with garlic for 24 h before UV irradiation (100 mJ/cm^2^) with a thin layer of PBS using UVB lamp (312 nm, Spectroline Model EB-160C, New York, NY, USA). In the preliminary study, UVB irradiation of keratinocytes led to a dose-dependent suppression of cell viability (69%, 50 mJ/cm^2^; 45%, 100 mJ/cm^2^; 19%, 150 mJ/cm^2^), compared with non-irradiated cells. A 100 mJ/cm^2^ irradiation dose was used in the following studies. After UVB irradiation, the cells were washed with warm PBS, and incubated with serum-free DMEM for 24 h. Mock-irradiated controls followed the same schedule of medium changes without UVB irradiation.

### 2.4. ROS Production

DCFH-DA was used to detect ROS production in cells. Cells which had been treated with garlic extract (50 and 100 µg/mL) prior to UV irradiation were incubated with 20 μM DCF-DA for 30 min, and harvested after 24 h. ROS formation was analyzed by a fluorometer (TECAN, SER-NR 94572, Salzburg, Austria) using 485 nm of excitation and 530 nm of emission filters. ROS production was expressed as a percentage of the fluorescence of non-UV irradiated control.

### 2.5. Quantitative Real Time RT-PCR

Total RNA, isolated using RNeasy^®^ Protect Mini kit (Qiagen, Valencia, CA, USA), was reverse transcribed to cDNA with the SuperScript First-Strand Synthesis System (Invitrogen, Eugene, OR, USA). The primer sequences for *MMP-1* were: forward, 5′-ATT CTA CTG ATA TCG GGG CTT TGA-3′; and reverse, 5′-ATG TCC TTG GGG TAT CCG TGT AG-3′. The primer sequences for *GAPDH* were: forward, 5′-TCA TCA ATG GAA ATC CCA TCA CC-3′; and reverse, 5′- TGG ACT CCA CGA CGT ACT CAG C-3′. PCR amplification was carried out using a QuantiTectTM SYBR Green PCR kit (Qiagen, Valencia, CA, USA). The PCR cycle was 94 °C for 10 min, followed by 40 cycles of reaction at 94 °C for 10 s, 58 °C for 15 s, and 72 °C for 20 s. The level of *MMP-1* mRNA was normalized to the level of *GAPDH*, and compared with a control (untreated sample) using the ΔΔCT method according to the manufacturer’s protocol. 

### 2.6. MMP-1 Production

Cells were cultured in 24-well plates (1 × 10^6^ cells/well), pretreated with garlic for 24 h, and exposed to UVB. The production of MMP-1 was determined using a commercial ELISA kit (Human total MMP-1 kit; R&D systems, Minneapolis, MN, USA). MMP-1 level was expressed as a percentage of non-UV irradiated control.

### 2.7. Cytokine Determination

HaCaT cells (5 × 10^6^/well) were pretreated with garlic for 24 h, and exposed to UVB. The concentrations of IL-1β and IL-6 in the culture supernatants were measured by human IL-1β and IL-6 ELISA kits purchased from R&D systems (Minneapolis, MN, USA) following the manufacturer’s instructions. Briefly, the culture supernatants of HaCaT cells were collected and added to the antibody coated wells. After 1 h incubation at 37 °C, the wells were washed with PBS-Tween 20 (pH 7.4) (Sigma-Aldrich, St. Louis, MO, USA), and alkaline phosphatase conjugated antibodies were added and incubated for another 1 h. The wells were washed, substrate solution was added, and incubated for another 1 h. The relative absorbance was measured at 450 nm and IL-1β and IL-6 concentrations were calculated using a standard curve.

### 2.8. Senescence-Associated β-Galactosidase (SA-β-gal) Histochemical Staining

Cells were cultured in 24-well plates (1 × 10^4^ cells/well), pretreated with garlic, and exposed to UVB. Cells were washed with PBS and fixed for 5 min in 3% formaldehyde. SA-β-gal staining was determined using a Senescence β-galactosidase staining kit (Cell Signaling Technology, Danvers, MA, USA). A total of 100 cells were counted in three randomized fields, and the percentage of blue stained senescence cells was counted using light microscope.

### 2.9. SIRT1 Activity

Cell extracts were obtained by using a lysis buffer (1% Triton X-100, 150 mM NaCl, 20 mM Tris, pH 7.5 with protease inhibitors mix, Sigma-Aldrich), and SIRT1 activities were determined by Sirt1 activity kit (CS 1040, Sigma-Aldrich) according to the manufacturer’s protocol. The fluorescence of the plate was determined with an excitation wavelength of 360 nm and an emission wavelength of 450 nm. SIRT1 activity was expressed as a percentage of non-UV-irradiated control.

### 2.10. Analysis of Organosulfur Compound

Analysis of the organosulfur compound of garlic extract was performed with an HPLC system (Agilent 110S, Waldbronn, Germany, UV detector). Compounds were separated on a µ-Bondapak C18 column (30 mm × 30 cm, Waters, MA, USA). The mobile phase consisted of methanol-water (70:30, *v*/*v*). Allicin (LTK Laboratories, Wako, Japan), diallyl disulfide and diallyl trisulfide (Sigma-Aldrich) were used as standards. HPLC was performed at a flow rate of 0.8 mL/min.

### 2.11. Statistical Analysis

Each experiment was performed in triplicate and all data are presented as mean ± SD. Significant differences between groups were analyzed by ANOVA followed by the Duncan’s multiple range test (*p* < 0.05). 

## 3. Results

### 3.1. Effect on Cell Free System Radical Scavenging Activity

**Figure 1:**
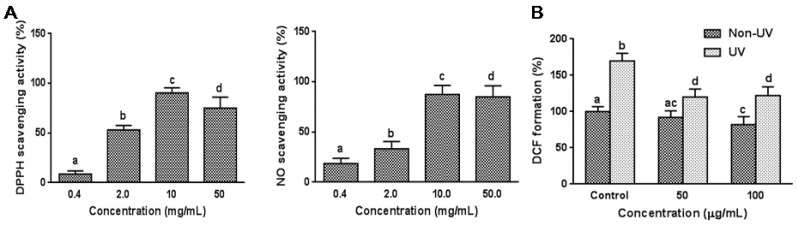
Antioxidant effects of garlic. (**A**) DPPH and NO radical scavenging activity of garlic extract in a cell-free system. The level of DPPH radical was measured spectrophotometrically at 515 nm. The NO scavenging capacity was assessed by Griess assay. The IC_50_ values for the DPPH radical and NO scavenging activities were 2.50 mg/mL and 4.38 mg/mL, respectively; (**B**) intracellular ROS levels induced by UVB were determined by the DCF–DA method. HaCaT cells, treated with garlic prior to UV irradiation (100 mJ/cm^2^), were incubated with 20 μM DCF–DA for 30 min, and harvested after 24 h. ROS formation was analyzed with a fluorometer (excitation; 486 nm, emission; 530 nm). Each bar represents the mean ± SD (*n* = 6). The bars with a different letter are significantly different from each other at the level of *p* < 0.05.

DPPH radical and NO scavenging activities are commonly used to evaluate antioxidative activities of various plants and pure compounds. The effect of garlic on free radical and NO scavenging capacities were determined in a cell free system. DPPH radical and NO scavenging activities were both increased sigmoidally with increasing garlic concentrations between 0.4 and 50 mg/mL, and DPPH and NO radical scavenging activity reached a saturation point at 10 mg/mL exhibiting 87.4 ± 9.0% and 90.4 ± 5.0% scavenging activity, respectively (Figure 1A). The effect of garlic on DPPH radical scavenging activity was greater than NO scavenging activity. The IC_50_ values for the DPPH radical and NO scavenging activities were 2.50 mg/mL and 4.38 mg/mL, respectively.

### 3.2. Effect on UVB-Induced ROS Generation in HaCaT Cells

Since intracellular ROS levels are known to increase in cells during cellular senescence [22], ROS generation in response to UVB-exposed HaCaT cells was determined using the 2′,7′-dichlorodihydrofluorescein diacetate (DCFDA)–ROS detection assay. The ROS formation was evaluated after cells were irradiated at 100 mJ/cm^2^ of UVB irradiation. As shown in Figure 1B, ROS generation was markedly increased in UVB-irradiated HaCaT cells compared with non-irradiated control cells, representing massive oxidant generation. Pretreatment with garlic significantly attenuated an increase (*p* < 0.05) in intracellular ROS content, which was triggered by UVB irradiation. The ROS level was quenched by 29.4% at 50 µg/mL garlic treatment compared with UVB-irradiated control reaching saturation point. The dose–response effect was not observed. In non-UV treated HaCaT cells, garlic treatment at 100 µg/mL concentration slightly reduced the ROS formation in (Figure 1B). Taken together, the results mentioned above indicate that garlic protects keratinocytes from UV-induced oxidative stress by scavenging various free radicals.

### 3.3. Effect on UVB-Induced MMP-1 Expression in HaCaT Cells

**Figure 2:**
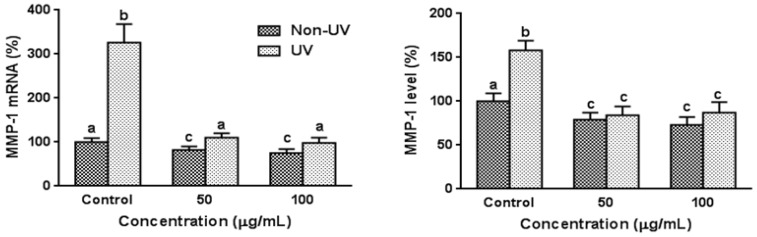
Effect of garlic on MMP-1 expressions in human skin keratinocytes. Cells were pretreated with garlic for 24 h prior to UVB irradiation (100 mJ/cm^2^) and harvested 24 h later. Expression of *MMP-1* mRNA was determined by quantitative real time RT-PCR. *GAPDH* was used as an internal control. MMP-1 level was determined using an ELISA kit. Data are expressed as the percentage of control (Non-UV) group. MMP-1 level of HaCaT cells before UV irradiation was 0.12 ± 0.02 pg/mL (100%). Each bar represents the mean ± SD (*n* = 3). The bars with a different letter are significantly different from each other at the level of *p* < 0.05.

The effects of garlic on expression of MMP-1 mRNA and protein were determined using quantitative real time RT-PCR and ELISA, respectively. Exposure of HaCaT cells to UVB significantly increased MMP-1 mRNA and protein expression by 3.26-fold and 1.58-fold, respectively, and pretreatment of garlic at 50 and 100 µg/mL reduced these MMP-1 expressions comparable to those of non-UV irradiated control cells (Figure 2). The dose–response effect was not observed. MMP-1 mRNA and protein expressions were slightly but significantly reduced by garlic treatment in non-UV irradiated control cells. 

### 3.4. Effect on UVB-Induced Pro-Inflammatory Cytokine Production in HaCaT Cells

**Figure 3:**
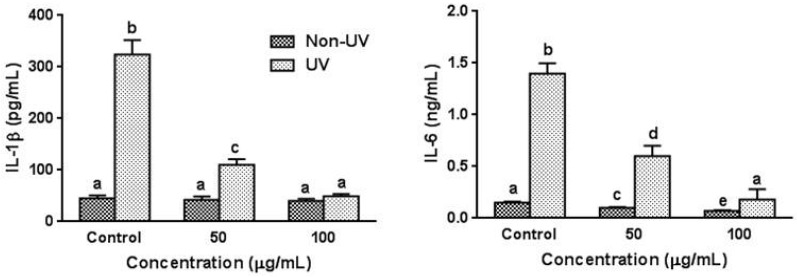
Effect of garlic on IL-1β and IL-6 productions in human skin keratinocytes. Cells were pretreated with garlic for 24 h prior to UVB irradiation and harvested 24 h later. The levels of IL-1β and IL-6 in culture media were determined by ELISA kit. Each bar represents the mean ± SD (*n* = 3). The bars with a different letter are significantly different from each other at the level of *p* < 0.05.

To assess the regulatory effect of garlic on the production of pro-inflammatory cytokines in UVB-irradiated HaCaT cells, the changes of IL-1β and IL-6 levels were investigated by the ELISA. As expected, UVB irradiation dramatically increased IL-1β and IL-6 levels in the absence of garlic by 7.2-fold and 9.3-fold, respectively, while the pretreatment of garlic significantly inhibited the UVB-induced elevation of IL-1β and IL-6 production in a dose-dependent manner (Figure 3). Pretreatment of garlic at 100 µg/mL concentration reduced these cytokine productions comparable to those of non-UV irradiated control cells. In non-UV exposed cells, pretreatment of garlic decreased the IL-6 level while the IL-1β level was not affected by garlic treatment. 

### 3.5. Effect on UVB-Induced SA-β-gal Activity in HaCaT Cells

**Figure 4:**
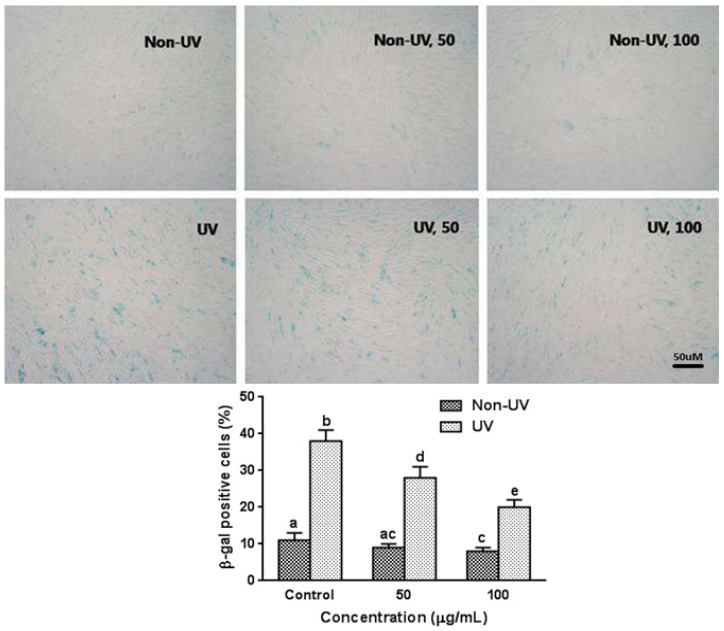
Effect of garlic on SA-β-gal activity in human skin keratinocytes. Cells were pretreated with garlic for 24 h prior to UVB irradiation (100 mJ/cm^2^) and cellular senescence was assessed by SA-β-gal activity staining. Each bar represents the mean ± SD (*n* = 3). The bars with a different letter are significantly different from each other at the level of *p* < 0.05.

The activity of the cellular senescence biomarker, SA-β-gal, in UVB-exposed HaCaT cells was examined by an SA-β-gal histochemical staining assay. The percentage of senescence cells in the non-UV irradiated HaCaT cells was low, and garlic treatment (100 µg/mL) slightly but significantly decreased SA-β-gal activity in non-UV exposed HaCaT cells (Figure 4). However, upon UVB irradiation, the percentage of SA-β-gal positive cells was increased in a dependent manner (data not shown). When cells were exposed to UVB (100 mJ/cm^2^), SA-β-gal activity markedly increased by 3.4-fold as compared with unexposed cells (Figure 4). Pretreatment with garlic at 50 and 100 µg/mL before UV exposure significantly inhibited the increase in SA-β-gal activity in a dose-dependent manner exhibiting 26.3% and 47.4% inhibition, respectively.

### 3.6. Effect on UVB-Induced SIRT1 Activity in HaCaT Cells

**Figure 5:**
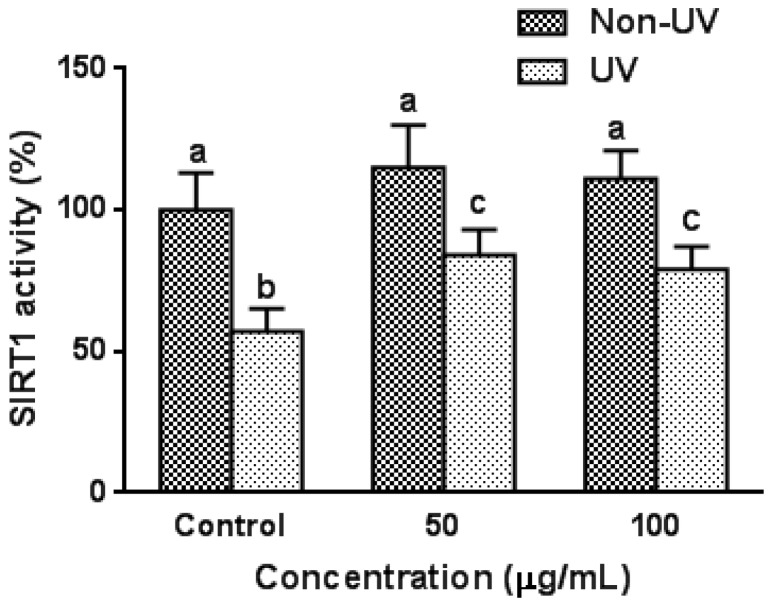
Effect of garlic on SIRT1 activity in human skin keratinocytes. Cells were pretreated with garlic for 24 h prior to UVB irradiation (100 mJ/cm^2^) and cellular senescence was assessed by SIRT1 activity. Data are expressed as the percentage of control (Non-UV) group (13.2 ± 1.8 unit/μg protein; 100%). Each bar represents the mean ± SD (*n* = 3). The bars with a different letter are significantly different from each other at the level of *p* < 0.05.

Since garlic treatment efficiently suppressed UV-induced increase in SA-β-gal levels in HaCaT cells, anti-aging effect of garlic was further investigated by measuring the SIRT1 activity, which has attracted attention as an anti-aging factor in recent years. As shown in Figure 5, the effect of garlic pretreatment on SIRT1 activity was not observed in non-UV irradiated control HaCaT cells. However, UV exposure suppressed SIRT1 activity by 43% compared to unexposed control cells, and pretreatment with garlic (50 µg/mL) significantly inhibited the UV-induced decrease in SIRT1 activity. The dose–response effect was not observed. The results suggest that garlic prevents UV-induced cellular senescence in terms of both SA-β-gal and SIRT1 activity.

### 3.7. Organosulfur Compounds in Garlic Extract

**Figure 6:**
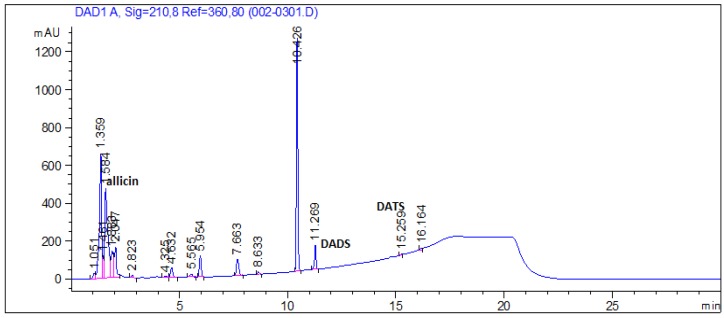
HPLC profile of garlic extract. Allicin, diallyl disulfide (DADS), and diallyl trisulfide (DATS) were evaluated.

Figure 6 shows the typical chromatogram profile of garlic extract used in the present study. Allicin, diallyldisulfide, and diallyl trisulfide, which have been reported as the most representative constituents of garlic, were determined. The concentrations of allcin, diallyldisulfide, and diallyl trisulfide were 141.9 ± 16.7, 1.6 ± 0.2, and 0.1 ± 0.01 mM, respectively. 

## 4. Discussion

As life expectancy increases in developed countries, the impact of aging on the function and appearance of skin is receiving growing interest. Skin aging is influenced by several factors, including genetics, environmental exposure (UV irradiation, xenobiotics, mechanical stress), hormonal changes, and metabolic processes (generation of reactive chemical compounds such as ROS) [23]. The influence of the environment, especially UV irradiation, is of considerable importance for skin aging. Skin aging due to UV exposure (photoaging) is superimposed on chronological skin aging. Although the typical appearance of photoaged and chronologically aged human skin can be readily distinguished, recent evidence indicates that chronologically aged and UV-irradiated skin share important molecular features including altered signal transduction pathways that promote MMP expression, decreased procollagen synthesis, and connective tissue damage [23]. This concordance of molecular mechanisms suggests that UV irradiation accelerates many key aspects of the chronological aging process in human skin.

The benefits of garlic to health have been proclaimed for centuries; however, only recently *Allium sativum* and its derivatives have been proposed as promising candidates for anti-aging agents. In 1994, Svendsen et al. [24] demonstrated that garlic has some youth-preserving, anti-aging and beneficial effects on human fibroblasts in terms of maximum proliferative capacity and morphological characteristics. The present study demonstrated that garlic exerted inhibitory activity against cellular senescence in UV-exposed human keratinocytes, HaCaT cells. Garlic pretreatment decreased senescence-associated SA-β-gal and SIRT1 activity, MMP-1 expression, and ROS generation in senescent keratinocyte cells induced by UV irradiation.

ROS have been reported to play a crucial role in the regulation of cellular senescence [2,3]. Intracellular ROS levels are elevated during both premature and replicative senescence, as well as UV-exposure, and treatment with antioxidants is known to repress cellular senescence [25]. ROS also activate the DNA damage signaling cascade, which induces cellular senescence [25]. Therefore, oxidative stress is a major risk factor underlying cellular senescence and photoaging [3,6], and the use of free radical scavengers to reduce the harmful effects of UV irradiation is a novel approach to photo-protection and skin aging prevention [14,15]. DPPH is a stable free radical donor that is widely used to test the free radical scavenging effects of natural antioxidants. NO is a free radical that reacts with oxygen to form oxides of nitrogen. NO and reactive nitrogen species (RNS) have been shown to be associated with common forms of skin diseases. NO liberated following UV irradiation plays a significant role in initiating erythema and inflammation [26]. NO can combine with UV-induced superoxide to form peroxynitrite, which exists in equilibrium with peroxynitrous acid. Several studies evaluated NO scavenging activity as an index of antioxidative and free radical scavenging activity. Boora et al. [27], Jagetia et al. [28], and Gaikwad et al. [29] evaluated NO and/or DPPH radical scavenging activities of plant extracts to examine natural antioxidant properties. In the present study, garlic exhibited antioxidant activity based on DPPH and NO free radical scavenging assay as well as ROS formation, implying that the inhibitory effect on cellular senescence might occur through its antioxidant activity. Garlic and its components have been widely accepted as an antioxidative agent [16,17,18,19]. Garlic contains vitamin C, selenium and β-carotene, which are well-known powerful antioxidants, as well as phytochemicals such as polyphenols. In addition, garlic contains many sulfur compounds (aliin, allicin, ajoene, diallyl disulfide, diallyl trisulfide, S-allylcysteine, vinyldithiines, S-allylmercaptocystein and others), and antioxidative effects of sulfur compounds such as allicin, allyl disulfide and allyl cysteine have been reported [19,30,31]. Furthermore, Li et al. [32] reported that the levels of organosulfur compounds, especially diallyl disulfide and diallyl trisulfide, were correlated with antioxidant capacities of garlic. However, the effect of the organosulfur compounds on skin aging related reports is limited. S-allyl cysteine, the most abundant component of aged garlic, exhibited antioxidative and anti-wrinkle activities [33]. Diallyl sulfide decreased UVB-induced thymine dimer-positive cells, terminal deoxynucleotidyl transferase-mediated dUTP nick end labeling, and NO levels in UVB irradiated hairless mice skin [34].

Alterations in collagen and elastin of the extra cellular matrix (ECM) are primarily responsible for the clinical manifestations of skin aging such as wrinkles, sagging, and laxity [35,36]. The atrophy of collagen and elastin fibers in skin aging is predominantly from the increased expression of their degradative enzymes, and collagen-degrading MMP-1 is upregulated and serves as the primary MMP in UV-exposed skin [14]. Collagenolytic MMP enzymes attack fibrillar collagen and elastin responsible for the dermal strength and resiliency. UV-induced ROS triggers complex signaling pathways including MMP over-expression and degradation of ECM in connective tissues [27,35]. Therefore, MMP-1 inhibitors have been identified as potential therapeutic agents that protect against photoaging and wrinkle formation. The results of the present study clearly demonstrated that garlic treatment significantly attenuates MMP-1 protein as well as mRNA expressions comparable to non-UV exposed control level. In a preliminary study, similar results were observed in HS68 fibroblasts (data not shown). Kim et al. reported that topical treatment of S-allyl cysteine downregulates MMP-3, -9, and -12 protein expressions [33]. Recently, we have reported that garlic supplementation (1% of the diet) in hairless mice prevented UV-induced ROS generation, lipid peroxidation levels, MMP-1 protein and mRNA expressions, procollagen mRNA expression, and wrinkle formation in hairless mice skin [37]. Furthermore, antioxidant enzyme activities were enhanced by garlic administration. These in vivo results further support the present in vitro results. 

Apart from shortened or dysfunctional telomeres, cells undergoing senescence are also associated with over-expression of inflammatory cytokines such as TNF-α, IL-1β, and IL-6 due to an age-related redox imbalance [4,5]. Several studies have demonstrated that high plasma levels of IL-6 are correlated with greater disability, morbidity, and mortality in the elderly [38]. In addition, high levels of IL-6, IL-1β and C-reactive protein are significantly associated with disease conditions in older individuals [39]. The constitutive productions of cytokines and other soluble factors are low in human keratinocytes, but various stimuli such as UV irradiation, can trigger the expression of pro-inflammatory cytokines. ROS produced by UV irradiation act as a second messenger in the signaling pathways that plays an important role in inflammation [40]. UVB irradiation induces the expression of the NF-κB, which induces the expression of several kinds of pro-inflammatory cytokines that may lead to photo-damage by regulating the expression of the members of the MMP family [41]. Evidence indicates that IL-1α, IL-1β, IL-6, and TNF-α play the most important roles in the over-expression of MMP-1 induced by UVB irradiation [7]. Furthermore, these cytokines are also involved in immune regulation and cell survival. Therefore, the present study investigated the influence of garlic on the expression of IL-1β and IL-6 in UVB-irradiated HaCaT cells. The results revealed that the levels of these cytokines were markedly reduced in the cells treated with garlic after UV irradiation in a dose-dependent manner. Prevention of inflammation using anti-inflammatory compounds, including cytokine generation inhibitors, appears to be one strategy assuaging photoaging. Antioxidant polyphenols has been shown to suppress the UV-induced pro-inflammatory cytokines of IL-1β and IL-6 in the dermis [42]. The pro-inflammatory cytokine inhibition effect of garlic observed in this study is consistent with that in the previous studies on the regulation of cytokines and chemokines exerted by garlic organosulfur compounds. The unstable precursor organosulfur compound allicin was found to inhibit spontaneous and TNF-α-induced secretion of pro-inflammatory cytokines and chemokines from intestinal epithelial cells [43]. Diallyl disulfide, diallyl trisulfide, and S-allyl cysteine were discovered to attenuate lipopolysaccharide-induced inflammatory mediators by down-regulating the NF-κB and MAPK signaling pathways [33,44]. 

The biomarkers of cellular senescence triggered by UVB examine permanent cell cycle arrest, enlarged and flattened cell morphology, and SA-β-gal activity [7]. In culture and in vivo biopsy, SA-β-gal activity has been measured in a variety of cells and tissues to demonstrate the onset of cellular senescence [7,45]. In the present study, treatment with garlic inhibited the increase in the SA-β-gal activity of UV-exposed HaCaT cells, which confirms that it plays an important role in the retardation of the accumulation of senescent keratinocytes in human skin. 

SIRT1 is the most well studied member of sirtuin family that has been shown to be linked to cell longevity through a number of biological functions such as gene silencing, apoptosis, oxidative stress and regulation of cellular life span [8,10]. SIRT1 expression levels reportedly decreased with cellular senescence [46] and forced SIRT1 expression promotes cell proliferation and inhibits cellular senescence [47]. ROS has been shown to reduce SIRT1 expression levels, suggesting that cellular senescence was induced by oxidative stress [48]. SIRT1 also has been reported to play an important role in UV response including UV-induced keratinocyte cell transformation. UV radiation downregulate SIRT1 in a time- and dose-dependent manner, and ROS-mediated JNK activation is involved in this SIRT1 down-regulation [15]. Furthermore, resveratrol, which has been considered as an important antioxidant, protects against UV-induced cell death [49]. Since, in the present study, garlic efficiently reduced UV-induced increase in SA-β-gal levels in HaCaT cells, the anti-senescence effects was further analyzed by measuring the activity of SIRT1. When HaCaT cells were exposed to UVB, SIRT1 activity was significantly decreased compared with unexposed cells (Figure 5). Pretreatment with garlic significantly inhibited the UVB-induced decrease in SIRT1 activity, suggesting that garlic protects keratinocytes from oxidative stress and cellular senescence in terms of both SA-β-gal and SIRT1 activity. Similar inhibition of UVB-induced decrease in SIRT1 activity with garlic treatment was observed in HS68 fibroblasts (data not shown). Recently, Ohguchi et al. reported that SIRT1 exerts a negative regulatory role in the production of MMP-1 and MMP-3 in human dermal fibroblasts [49]. Treatment with a potent inhibitor of SIRT1 increased the basal expression levels of MMP-1 proteins, and knockdown of endogenous SIRT1 led to increased expression of MMP-1 at both mRNA and protein levels. Therefore, the effect of garlic on increased SIRT1 activity may be affected, at least in part, by the decreased expression of MMP-1.

## 5. Conclusions

The present study provides the first evidence that garlic protected HaCaT keratinocytes from UV-induced cell damage and cellular senescence as determined by expression of SA-β-gal and SIRT1 activity. The presence of garlic scavenges UVB-induced free radicals and reduces ROS generation in HaCaT keratinocytes, which, in turn, inhibits MMP-1 expression, pro-inflammatory cytokines productions, and the activities of senescence related biomarkers. Based on these characteristics, garlic could have good potential as an anti-aging material. However, it is important to note that the results of this study were based on experiments conducted using an in vitro cellular model. Accordingly, further studies using in vivo animal models should be conducted to elucidate the mechanism by which garlic prevents cellular senescence and demonstrates its inhibitory effects.
